# IgE repertoire and immunological memory: compartmental regulation and antibody function

**DOI:** 10.1093/intimm/dxy048

**Published:** 2018-07-20

**Authors:** Hannah J Gould, Yu-Chang Bryan Wu

**Affiliations:** 1 Randall Centre in Cell and Molecular Biophysics, King’s College London, London, UK; 2 MRC Asthma UK Center in Allergic Mechanisms of Asthma, London, UK

**Keywords:** allergy, IgE repertoire, immunological memory, mucosa, next-generation sequencing

## Abstract

It is now generally recognized that bone marrow is the survival niche for antigen-specific plasma cells with long-term immunological memory. These cells release antibodies into the circulation, needed to prime effector cells in the secondary immune response. These antibodies participate in the surveillance for antigen and afford immune defence against pathogens and toxins previously encountered in the primary immune response. IgE antibodies function together with their effector cells, mast cells, to exert ‘immediate hypersensitivity’ in mucosal tissues at the front line of immune defence. The constant supply of IgE antibodies from bone marrow plasma cells allows the rapid ‘recall response’ by mast cells upon re-exposure to antigen even after periods of antigen absence. The speed and sensitivity of the IgE recall response and potency of the effector cell functions are advantageous in the early detection and elimination of pathogens and toxins at the sites of attack. Local antigen provocation also stimulates *de novo* synthesis of IgE or its precursors of other isotypes that undergo IgE switching in the mucosa. This process, however, introduces a delay before mast cells can be sensitized and resume activity; this is terminated shortly after the antigen is eliminated. Recent results from adaptive immune receptor repertoire sequencing of immunoglobulin genes suggest that the mucosal IgE^+^ plasmablasts, which have undergone affinity maturation in the course of their evolution *in vivo*, are a source of long-lived IgE^+^ plasma cells in the bone marrow that are already fully functional.

## Introduction: immune memory of IgE responses and their effector cells

Immunological memory is the capacity to mount a recall response to an antigen after a prolonged absence of antigen, given the rapid turnover of antibodies *in vivo*. Such memory may last for the lifetime of an individual (1, 2). It is embodied in selected populations of memory B cells and plasma cells, endowed with long-term survival compared with their precursors that have half-lives of only a few days or weeks.

The vast majority of B cells undergo programmed cell death (apoptosis) and are regenerated as mature naive B cells from the bone marrow that compete for selection into the memory pool on the basis of their affinity for antigen. The selected cells differentiate into memory cells or plasma cells. The memory B cells act in the surveillance for antigen in the circulation and mucosal tissues. At sites of exposure to antigen, they proliferate and differentiate into antibody-secreting plasma cells. The majority of plasma cells are short-lived, but some succeed in migration to the bone marrow, which provides a survival niche. These cells secrete antibodies into the circulation that sensitize effector cells to antigen in all parts of the body.

The constant supply of IgE antibodies is critical for ‘immediate hypersensitivity’ in mucosal tissues at the frontline of immune defence. Binding of IgE antibodies to the high-affinity IgE receptor (FcεRI) on mast cells in tissues and basophils in the circulation ‘sensitizes’ the cells for an anaphylactic degranulation, which is characterized by the immediate and explosive release of pharmacologically active pre-formed mediators from storage granules and concurrent synthesis of inflammatory lipid mediators from arachidonic acid. Allergic reactions are triggered by the binding of multivalent allergens to the IgE (discussed below), thereby cross-linking the FcεRI receptors and initiating signal transduction leading to cell activation. The principle effects of these products are vasodilation and increased permeability and smooth muscle contraction, which are manifested in differing symptoms in rhinitis, asthma, atopic dermatitis, food allergy and other allergic diseases.

The speed and sensitivity of the mast cell reaction, triggered by allergens through IgE, exceed those triggered through other antibody classes. Moreover, the physiological effects of the products released upon antigen activation of mast cells are well adapted to resist bacterial infections and neutralize toxins in the environment, preventing further harm to the individual. Unfortunately, this enhanced effector function is too often mis-directed in man by IgE recognition of normally harmless antigens (allergens), leading to the development of allergic disease.

In this review, we discuss the natural history of IgE^+^ B cells, beginning with their origin in the primary immune response, the developmental pathway into long-lived allergen-specific plasma cells and homeostasis *in vivo*. Focusing on the IgE response to aeroallergens in the respiratory track, we speculate that the respiratory track mucosa is the site where the activity of IgE is optimized and where bone marrow plasma cells that establish the immune memory of aeroallergen-induced IgE responses originate.

## Development of preimmune B cells in the bone marrow

The capability of B cells to defend against a wide and ever-evolving array of pathogens is largely attributable to the unique diversity in their immunoglobulin repertoire. An antibody molecule, either expressed as the surface B-cell receptor (BCR) or in the secreted form of a soluble antibody, is composed of two identical kappa (κ) or lambda (λ) immunoglobulin light chains (IgL) plus two identical immunoglobulin heavy chains (IgH; five major isotypes in humans), each containing an N-terminal variable (V_κ_, V_λ_ or V_H_) region and a C-terminal constant (C_κ_, C_λ_ or C_H_) region. Combinatorial association between the variable regions (V_H_:V_κ_ or V_H_:V_λ_) forms the antigen-binding site of the antibody, and its effector function is determined by the expression of a specific C_H_ region that corresponds to the antibody isotype (or class)_._

**Fig. 1.**
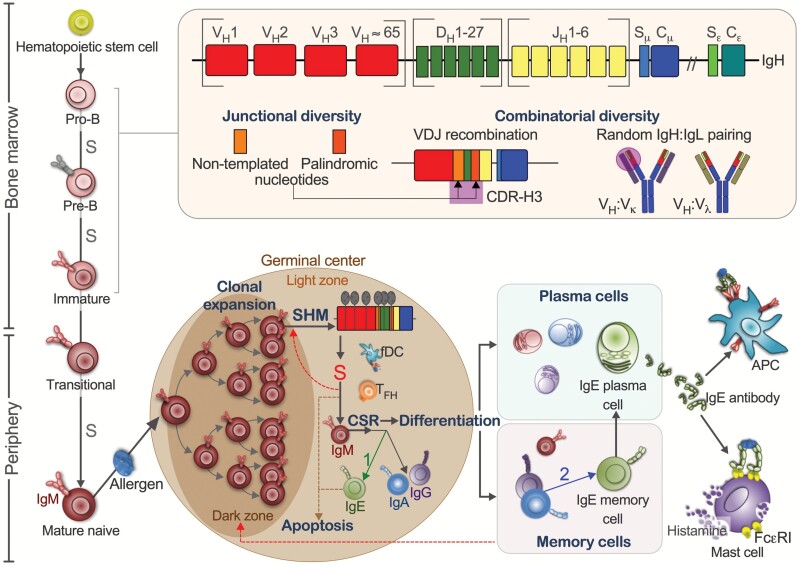
Development of the IgE^+^ B-cell repertoire. Top-left: the primary repertoire of early B cells in the bone marrow acquires combinatorial and junctional diversity (shown in the box) through V(D)J recombination (only IgH illustrated), random paring of IgH with IgL (λ or κ) chains and N/P nucleotide editing at the CDR-H3 junctions (corresponding to the purple circle). Bottom-left: following activation by allergen in the periphery, GC B cells in the dark zone undergo clonal expansion and SHM. In the light zone, GC B cells can undergo CSR to IgE in two pathways: (1: green arrow) direct switching from IgM^+^ precursors or (2: blue arrow) sequential switching via IgG^+^ or IgA^+^ intermediates; the second pathway may occur outside lymphoid tissues, such as the ectopic GC in mucosal tissues. Following antigen selection in the presence of TFH cells and fDCs in the GC light zone, B cells are selected to either cycle in the GC for further affinity maturation (the red dotted paths) or differentiate into memory B cells and antibody-secreting cells (plasmablasts→plasma cells); unselected B cells undergo apoptosis. Bottom-right: IgE antibodies secreted from plasma cells can sensitize mast cells or antigen-presenting cells (APCs), i.e. DCs via FcεI and B cells via FcεII or CD23 (not shown). Cross-linking of IgE via FcεRI on mast cells induces degranulation of mast cells and release of histamine, proteases and metabolites that collectively effect the symptoms of allergy. Four antibody isotypes are coloured red for IgM, blue for IgA, purple for IgG and green for IgE. Selection processes are indicated ‘S’ in red for affinity-based selection in the GC and ‘S’ in grey for selection against non-autoreactive functional BCRs of the preimmune repertoire.

During early ontogeny (Fig. 1), B cells achieve combinatorial diversity through V(D)J recombination, which requires recombination activating gene (RAG) proteins to assemble a complete V_κ_, V_λ_ or V_H_ exon from component germline variable (V), joining (J), and diversity (D; only in V_H_) gene segments. This is accompanied by imprecise insertion/deletion of N/P nucleotides at the junctions between the recombined gene segments, thus creating more genetic and protein structural variability of the variable region. These highly variable junctions, commonly termed complementarity-determining regions (CDRs), correspond to six hypervariable loops in the three-dimensional structure of an antibody that specifically interact with antigens. The third CDR of V_H_ (CDR-H3) has the greatest diversity of the six CDRs in amino acid residues, rendering it remarkably unique to each progenitor B cell that is subsequently passed on to all of its clonally expanded progeny. Indeed, CDR-H3 is routinely used to determine clonal lineages of B cells in studies of the antibody repertoire.

The combinatorial and junctional mechanisms occur in the absence of foreign antigens in the bone marrow and can, in theory, produce >10^8^ possible different antigen-binding sites exceeding the actual total number of B cells in a human. The recombined V_H_ exon in a developing B cell is then assembled with the most 5′ constant mu (Cμ) gene (closely linked to Cδ), allowing surface BCR expression of the IgM isotype (and IgD; not discussed herein). Naive (CD27^–^IgM^+^IgD^+^) B cells are identified as new (bone marrow) emigrant B cells that have completed cell maturation and passed the peripheral checkpoint against autoreactivity. Outside the bone marrow, mature naive B cells undergo phenotypic differentiation and further diversify their immunoglobulin repertoire in response to antigen challenge.

## Antigen-driven immunoglobulin diversification and affinity maturation

Following antigen activation, activation-induced cytidine deaminase (AID) initially in naive B cells mediates class-switch recombination (CSR), which displaces the IgM constant region (Cμ) for the expression of a secondary isotype (IgG, IgA or IgE) and sub-class (IgG_1,2,3,4_ or IgA_1,2_), ultimately diversifying the antibody effector functions without altering antigenic specificity. The germline C_H_ genes (Cγ_3_, Cγ_1_, Cα_1_, Cγ_2_, Cγ_4_, Cε or Cα_2_) that encode non-IgM isotypes (IgG_3_, IgG_1_, IgA_1_, IgG_2_, IgG_4_, IgE or IgA_2_) lie downstream to Cμ in a tandem array on human chromosome 14. Preceding each C_H_ gene, a specific stretch of repetitive DNA, termed switch (S; e.g. Sμ, Sγ_3_, Sε, etc.) region, instructs AID activity to initiate CSR. Isotype switching from IgM requires AID to deaminate cytidines to uridines within the (donor) Sμ and a downstream (acceptor) S region DNA; this leads to DNA cleavage at the deaminated residues, followed by DNA recombination between donor and acceptor S regions. Excision of switch circles by looping-out deletion of any intervening DNA subsequently enables the expression of a secondary C_H_ gene with the rearranged VDJ genes. Further CSR can occur in a B cell between the newly expressed C_H_ and any remaining (farther downstream) germline C_H_ genes, upon interaction of its BCR with an antigen of the same specificity.

Isotype switching is often accompanied by somatic hypermutation (SHM), a diversification process that contributes to a large spectrum of antibody affinity. SHM is initiated by AID in stimulated B cells, which introduces point mutations into the recombined V(D)J genes, typically targeting certain hotspots (e.g. RGYW motifs) within CDRs ultimately to modify (and improve) the structure of the antigen-binding site. This, together with CSR, generally occurs in the germinal centres (GCs) of B-cell follicles in constitutive lymphoid tissues. Following antigen activation, B cells undergo cell division and clonal expansion, leading to the formation of two GC microanatomical compartments: the dark and light zones. Proliferative B cells diversify their Ig repertoire through SHM in the dark zone, followed by antigen selection in the light zone where clonal members compete for antigen presentation to T follicular helper (T_FH_) cells in the presence of follicular dendritic cells (fDCs) – a crucial step in affinity maturation of functional antibodies (3). Selected GC B cells can undergo CSR in the light zone or re-cycle through the dark zone for further immunoglobulin modifications. After phenotypic differentiation, B cells exit the GC as memory (IgD^–^CD27^+^) B cells or plasmablasts (CD19^+^CD27^hi^CD38^hi^CD138^−^) that may eventually join the long-lived (CD19^–^CD27^hi^CD38^hi^CD138^+^) plasma cell population in the bone marrow.

IgE, together with IgG and IgA, is one of the three ‘switched’ isotypes resulting from CSR. IgE switching can occur directly from IgM (IgM→IgE) or sequentially through one or more of the prior-switched intermediates (IgM→IgG_3_→IgG_1_→IgA_1_→IgG_2_→IgG_4_→IgE); IgE may switch to IgA_2_ by replacing Cε with the last germline Cα_2_ gene segment. Since affinity maturation can occur in the precursors of IgE^+^ B cells, the pathway of IgE switching has significant implications for IgE affinity to antigen: direct switching to IgE from an IgM^+^ precursor may result in low IgE affinity and apoptosis of GC B cells during selection (4), whereas sequentially switched IgE can ‘inherit’ a higher affinity from the hypermutations previously selected in IgG^+^/IgA^+^ precursors (5).

## Antigens and IgE antibodies for effector function

The minimal requirement for mast cell activation is the cross-linking of its high-affinity IgE receptors, FcεRI molecules, on the surface of the mast cell. This is normally effected by antigen or allergen binding to IgE–FcεRI complexes on the mast cell and makes stringent demands on the antigen. It has been shown that the spacing between the two adjacent complexes is critical (6) and there are undoubtedly topological complexities that are not yet fully understood. Unlike antibodies of other isotypes, IgE can assume an acutely bent conformation (7), which may lead to steric interference from the cell in the binding of certain epitopes. Antibodies of the five different isotypes have different ‘reach’, depending on the length and flexibility of their hinge regions. Thus, the degree of specificity for antigen binding may vary between antibodies of different isotypes.

**Fig. 2.**
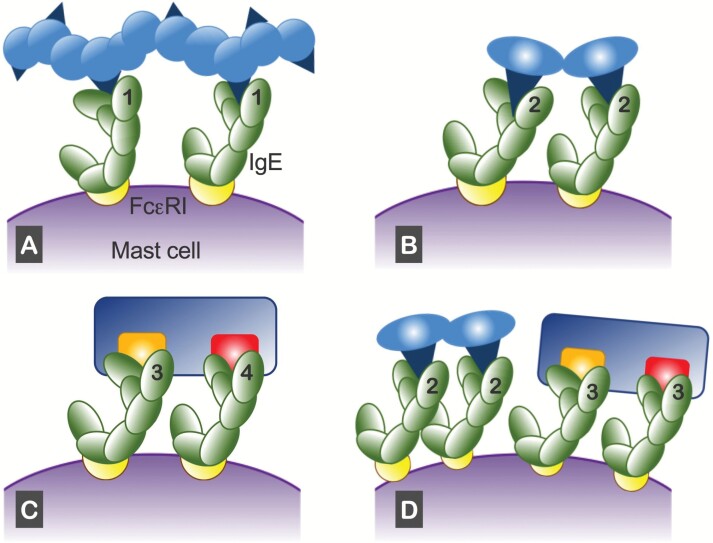
Models of IgE cross-linking on mast cells. Two IgE molecules on FcεRI receptors attached to a mast cell can be cross-linked by (A) a single antigen with multiple identical epitopes (i.e. ‘1’ and ‘1’ labelled on the antigen-binding sites), (B) an oligomeric antigen (e.g. a homodimer) with identical epitopes on the subunits, (C) a single antigen with two different epitopes and (D) two different antigens with different epitopes (e.g. combining mechanisms C and D). Four IgE specificities are indicated by numbers (1–4) on the antigen-binding sites.

Nevertheless, certain general rules apply to the cross-linking of FcεRI on mast cells and basophils by allergens. Cross-linking can occur under the following circumstances:

1. Antigens, such as certain pathogens, polysaccharide chains or DNA molecules, may contain several identical epitopes, which bind to IgE molecules of the same specificity (Fig. 2A).
2. Antigens with a single epitope may form non-covalently linked oligomers (homodimers, trimers, etc.), each of which binds to IgEs of the same specificity (Fig. 2B).
3. Antigens may contain at least two different epitopes, disposed in a manner that allows simultaneous attachment to IgEs of the corresponding specificities (on the same mast cell). Such antigens may be particularly efficient in cross-linking IgEs on mast cells (Fig. 2C). The multiple epitopes on the peanut Ara h2 allergen, described below (8), may allow it to initiate an allergic reaction.
4. There may be more than one allergen in allergenic materials, such as grass pollen in combination with house dust mite (HDM) faecal pellets. Each may satisfy one of the above criteria (Fig. 2A–C) and form separate complexes on the same mast cell, contributing additive effects on the strength of mast cell activation (Fig. 2D).

In addition, there are requirements of the allergen-specific IgE antibodies, as comprehensively investigated by Christensen *et al*. (9). They comprise total IgE concentrations, concentrations of allergen-specific IgE relative to non-allergen-specific IgE, concentration uniformity among individual allergen-specific IgE clones, IgE affinity for allergen and clonal diversity. The interplay of these factors affects the severity of allergic responses.

## Regulation of IgE and homeostasis

IgE is the least abundant of the five antibody isotypes (IgM, IgD, IgG, IgA and IgE); the serum concentration of IgG is about 10000 times higher than that of IgE in non-allergic subjects (20–30 versus 0.001 mg ml^−1^). Although it is highly variable, the concentration of IgE is only on average three times higher in allergic individuals. In light of its inflammatory and potentially adverse effects, i.e. uncontrolled mast cell activation or basophil activation that can lead to life-threatening systemic anaphylaxis or acute severe asthma exacerbations, a wide variety of immunoregulatory mechanisms are necessary to suppress IgE expression (10, 11).

It is evident from the low frequency of IgE switching (12) and poor survival of nascent IgE^+^ B cells (5, 13, 14) that the expression is generally suppressed to restrict the immune repertoire of IgE^+^ B cells in the system. This occurs at many stages in the lifetime of IgE^+^ B cells and can be observed following antigen stimulation (15), phenotype and clonal lineage determination (5, 16) and chemokine-directed cell migration toward specific anatomical compartments (17). Unlike other antibody classes, membrane IgE (as BCRs) remains active for cell signalling even in the absence of allergen (14, 18), but its activity can be regulated by soluble CD23 (19). It is expected that the above factors may be exploited for the discovery of new targets for clinical intervention in allergic disease.

Secreted IgE has a short half-life in mucosal tissues of ~2 weeks *in situ*, in spite of the fact that IgE dissociates from its high-affinity receptor more slowly than any other isotypes from their corresponding receptors (20). Yet, immediate hypersensitivity can be maintained for decades after initial allergen exposure because of the IgE^+^ memory plasma cells that reside in the bone marrow: IgE released into the periphery from bone marrow may be actively captured by mast cells in mucosal tissues (21) and/or transported into the mucosa by other effector cells expressing low-affinity IgE receptors, FcεRII (CD23). Under equilibrium conditions *in vivo*, mast cells in tissues have been shown to remain permanently sensitized to allergen. Upon renewed allergen stimulation, IgE is synthesized *de novo* by memory B cells that undergo IgE switching and differentiation into IgE^+^ plasma cells (13, 14, 22). The delay, however, would not support IgE-mediated immediate hypersensitivity, highlighting its unique dependence on long-lived IgE^+^ plasma cells in the bone marrow.

## The origin of IgE immune memory

Evidence that bone marrow is the repository of allergic memory was at hand in 1919, well before the discovery in 1961 of IgE. A clinical case study described a non-allergic patient who, after a bone marrow transplant from a horse-allergic donor, suffered an asthma attack while riding a horse in Central Park, New York (23). This report, and later studies of transplant-acquired allergies (24), did not identify the cell populations that transferred IgE immune memory. Such deficiency was addressed much later using mouse models for adoptive transfer of B cells (4, 13).

As demonstrated by Talay *et al*. (13), IgE^+^ memory (B220^+^IgD^–^GL7^–^CD38^hi^) B cells adoptively transferred from immunized (human M1/GFP) mice induced a serum IgE response to the recall antigen in naive RAG^–/–^ recipients. Using a different transgenic CεGFP mouse (TBmc or BALB/c mice with CεGFP) system, He *et al*. later reported that IgE^+^ cells (plasma cells and B220^+^CD138^–^ GC B cells) generated during the primary immunization were associated with direct IgE switching and lower SHM rates, expected to have low IgE affinity and offer only transient protection (4). He *et al.* proposed that immune memory of IgE responses was restricted to the plasma cell lineage in this mouse model; this depended on the transferred IgG^+^ GC B cells to undergo sequential switching to IgE to differentiate into long-lived IgE^+^ plasma cells following the secondary immunization in recipient mice. The same characteristics may hold for the human system: antibody secretion by IgE^+^ plasma cells transiently present in the peripheral circulation alone, assayed by the *in vitro* incubation of peripheral blood mononuclear cells, was judged to be insufficient to maintain the memory of IgE responses (25, 26). Although IgE^+^ memory (IgD^–^CD27^+/-^) B cells have been reported in man, their functions and cell fate remain unclear (27). In addition to the bone marrow, we and others have regarded the mucosal tissues of target organs as a peripheral source of IgE immune memory in asthma and allergy.

## Local IgE repertoire in the respiratory tract mucosa

Early clinical studies further demonstrated that the IgE-secreting plasma cells are present in the nasal mucosa in patients with allergic rhinitis (AR) (28–30). It was shown that a sub-group of patients allergic to grass pollen, who had negative skin prick tests and undetectable levels of allergen-specific lgE antibody in sera, had high titres of the antibodies against the allergens to which they reacted in their nasal secretions; this was the first evidence for local IgE antibody production and activity in the respiratory tract mucosa (31).

Later work supported this conclusion by immunohistochemistry staining of nasal mucosal tissues, showing an increase in the IgE^+^ plasma cells in seasonal AR patients compared with healthy controls (30). *De novo* synthesis and secretion of IgE protein in the mucosa were confirmed by incubating nasal biopsies *ex vivo* with radioactive amino acids and showing increased amounts of radioactive IgE in the medium as a function of time (32). The proportion of total IgE that was grass pollen- or HDM-specific IgE ranged up to 50% in this *ex vivo* system, an invariably higher proportion than found in the circulation of the same individual, where it was sometimes undetectable. We calculated that a hundred times more IgE was produced than required to saturate all the IgE receptors on mast cells in the tissue (10); thus, the excess IgE must spill out into the circulation and the nasal secretions.

Switch circles are the deleted by-products during CSR, containing the looped-out germline C_H_ genes and a switch junction recombined from the donor (3′ of the cleave site) and acceptor (5′ of the cleave site) S regions. In IgE^+^ B cells directly switched from IgM, the donor Sμ region is partially retained in switch circles and spliced to the acceptor Sε region to form one Sε-Sμ junction in switch circles; similarly, the Sμ donor can be joined to an Sγ acceptor as one Sε-Sγ junction if IgM switches to IgG. For sequential switching to IgE from the IgG that has descended from IgM, switch circles contain either Sε-Sγ or Sε-Sμ-Sγ junctions; this depends on whether AID cleaves the Sγ or Sμ portion of the Sμ-Sγ donor (a hybrid S region resulted from IgM to IgG switching) before being recombined with the acceptor Sε region. In any case, the presence of Sγ DNA (or similarly Sα_1_) in switch circles provides a minimum estimate of sequential switching.

We and others have used switch junctions (Sε-Sγ or Sε-Sμ in switch circles) as molecular markers for local isotype switching to IgE in the bronchial mucosa of asthma (33) and in the nasal mucosa of AR (34, 35). Sε-Sγ_4_ junction DNA was detected at the highest frequency in switch circles after the *ex vivo* allergen incubation of the nasal biopsies from AR patients sampled out of pollen season (35). This highlighted affinity maturation of the IgG_4_ precursors with prior allergen exposure and sequential switching to IgE in local mucosal tissues as the important process of IgE development in seasonal AR. As the last switch among the four IgG subclasses, IgG_4_ has maximum opportunity to have undergone affinity maturation before competing with IgE for antigen; this may be relevant to observations in immunotherapy discussed below. As expected from active GC reactions (SHM and CSR) and the presence of AID transcripts, clusters of B cells (potentially precursors of ectopic GCs) were observed in the mucosal tissues (35).

## Migration of mucosal plasma cells to the bone marrow

To test whether the nasal mucosa is the direct source of IgE^+^ plasma cells in the bone marrow, Luger *et al*. (15) immunized mice with ovalbumin and then challenged them with oral inhalation of an ovalbumin-containing aerosol. At varying times thereafter, the mice were treated with cyclophosphamide to kill dividing B cells, while sparing the plasma cells in bone marrow that have ceased dividing. While mice were exposed to ovalbumin, B cells proliferated into plasma cells of IgG_1_, IgA and IgE isotypes in the lung and the animals developed airway inflammation. Plasma cells from the mucosa joined the pool of ovalbumin-specific plasma cells in the bone marrow and became resistant to cyclophosphamide. Termination of ovalbumin inhalation depleted ovalbumin-specific plasma cells from the lungs, while preserving them in the bone marrow. This incisive experiment proves that the respiratory mucosa is a direct source of aeroallergen-directed IgE responses in the bone marrow of mice and confirmed that the bone marrow is the repository for IgE immune memory.

## A new step forward

Mechanisms involved in the development of B cells can be investigated by tracing the molecular footprints of GC reactions in the corresponding immunoglobulin sequences. Our early immunoglobulin gene profiling study was the first to reveal *in situ* intraclonal SHM in mucosal B cells in AR by Sanger sequencing of 120 immunoglobulin gene sequences (36).These clones were likely to represent only a miniscule fraction of the entire mucosal B-cell repertoire. We and others have since characterized local B cells more comprehensively, using massively parallel next-generation sequencing (NGS) of the adaptive immune receptor repertoire (AIRR).

## AIRR sequencing of B cells

The advent of AIRR sequencing (AIRR-Seq) technologies has revolutionized our capacity to study the clonal evolution of B cells in adaptive immunity (37–40). AIRR-Seq affords an analytic power of the IgE repertoire several orders of magnitude greater than Sanger sequencing, thus revealing previously unattainable knowledge concerning this rare and important antibody class (Table 1). This is exemplified here by three pioneering studies of IgE repertoires in allergic disease (8, 41, 42).

**Table 1.** Next-generation and Sanger sequencing analysis of IgE repertoires in allergic diseases

Allergy (ref.)	Cohort size	Sequencer	IgE (total) reads	Specimen	Specificity	Key findings
Aeroallergy (8)	16 adults: 1-year SIT (with/without) = 8/8	NGS (Roche 454)	90241 (594364)	PB, NB	scFv phage display (52 IgE clones)	*in situ* CSR & SHM
Seasonal AR (41)	10 adults: HC = 3, AR (in/out season) = 3/4	NGS (Roche 454)	8135 (97610)	PB, NB	–	*in situ* CSR & SHM
Peanut allergy (42)	27 OIT subjects (4–43 years)	NGS (Illumina MiSeq)	Unknown (>100000 per subject)	PB	mAb expression (5 IgE clones)	IgE–IgG/A clonality
Nut allergy, aeroallergy (47)	31 adults: allergic = 9, HC = 24	NGS (Illumina MiSeq)	175585 (15843270)	PB	–	IgE more related to memory cells
Peanut allergy, bee allergy (48)	10 subjects: peanut/ bee = 6/4	NGS (Roche 454)	31248 (53688)	PB	–	Antigen selection of IgE
Seasonal AR (49)	6 adults	NGS (Illumina MiSeq)	7499998 (31461115)	PB, BM	–	Diverse BM IgE repertoire
Aeroallergy (50)	6 adults	Sanger	296	PB	–	Restricted IgE repertoire
AA (51)	1 adult	Sanger	10 (41)	PB	–	IgE V_H_5 usage bias
AA, AD (52)	3 children	Sanger	50	PB	–	Restricted IgE repertoire
Seasonal AR (53)	3 adults	Sanger	51	PB, NB	–	Oligoclonal IgE
Aeroallergy (54)	1 adult	Sanger	51	PB	phage display	Polyspecific IgE
AA, AD (55)	13 children	Sanger	1366	PB	–	Superantigen activation
AA, AD (56)	14 adults	Sanger	177 [V_H_3]	PB	–	IgE clonal expansion
Seasonal AR (57)	17 adults: AR = 13, HC = 4	Sanger	51	PB, NB	–	Superantigen activation
AA (58)	1 adult	Sanger	30	BB	–	*in situ* CSR & SHM
Rhinosinusitis (59)	11 adults	Sanger	195	Sinus	–	*in situ* SHM

AA, allergic asthma; AD, atopic dermatitis; BB, bronchial biopsy; BM, bone marrow; HC, healthy control; NB, nasal biopsy; PB, peripheral blood.

### Influence of seasonal exposure to grass pollen on local and peripheral blood IgE repertoires in patients with allergic rhinitis

In 2014, we reported the first AIRR-Seq profiling of the mucosal IgE^+^ B cells in the context of seasonal AR (41). Large-scale analysis of VDJ-C_H_ genes in this study revealed intraclonal diversification of B cells through SHM and CSR, as well mutational differences in V_H_ associated with isotypes (IgA, IgG, IgM and IgE), compartments (blood and nasal mucosa) and allergic status (healthy, AR outside season and AR in season).

For IgE^+^ clones, the highest V_H_ mutational level and strongest evidence of antigen-driven selection were observed in AR subjects, particularly in nasal biopsies sampled during the pollen season. The study did not address the associated cell phenotypes, as in our unpublished work, or allergen specificity, elucidated for the first time in the two other studies featured below. But it demonstrated seasonal effects of natural allergen exposure on SHM activities and enrichment of high-affinity IgE locally in the nasal mucosa in AR. This was also reflected in the increased diversity of the mucosal IgE repertoire in AR during the pollen season, which might generate new paratopes, thereby increasing the disease severity as seen in the peanut allergy study (8). It must be noted that tissue-specific homing of pollen-stimulated B cells to the nasal mucosal tissues from elsewhere is an alternative explanation for these observations.

In addition to IgE compartmental clonality, we discovered that the clonal relationship between IgE and IgG/IgA variants was restricted to the AR subjects. This, along with similar V_H_ mutational levels between the three isotype-switched repertoires, suggested that sequential IgE switching from isotype-switched precursors plays an important process in the pathogenesis of AR.

### Single cell B-cell deconvolution of peanut-specific antibody responses in allergic patients

By integrating NGS with the monoclonal antibody (mAb) technology, Ho *et al.* (42) provided the first AIRR-Seq study on the evolution of peanut-specific IgE^+^ cells during oral allergen immunotherapy (OIT). Specific B cells for two major peanut allergens, Ara h1 or Ara h2, were identified in the blood by flow cytometry and subsequently expressed as IgG_1_ mAbs, capable of recognizing a diverse population of conformational and/or linear epitopes. The Ara h2-specific mAbs, for example, recognized multiple linear epitopes in a 24-amino acid peptide array; two of the corresponding IgEs against these epitopes may suffice to initiate an allergic response (Fig. 2C).

Peanut-specific mAbs were clonally aligned with hypermutated IgG_2_, IgG_4_, IgA or IgE variants in the AIRR-Seq data, as an indication of elevated SHM activities during OIT. These clones lacked intraclonal relatedness between IgM and IgE isotypes, in line with the hypothesis that the reactivity of IgE^+^ B cells to peanut allergens depends on sequential IgE switching and affinity maturation of isotype-switched precursors. Furthermore, the detection of peanut-specific IgG_4_ clones that were hypermutated was consistent with the putative IgE-blocking activity of IgG_4_, potentially with enhanced efficacy, during OIT (43).

Overall, the report by Hoh *et al*. this report pointed to OIT-induced changes in the cellular and cytokine responses (e.g. involving helper T cells) that might re-direct the pathway of isotype switching from IgE to IgG_4_ in peanut-specific B cells (44). Whether this occurred within the target organ of peanut allergy, i.e. gut mucosa, remains to be investigated.

### Persistence and evolution of allergen-specific IgE repertoires in specific allergen immunotherapy

Levin *et al.* (8) carried out a longitudinal AIRR-Seq profiling of allergen-specific B cells in subjects undergoing specific allergen immunotherapy (SIT). In this study, specific allergens were administrated subcutaneously (differing from the oral route in OIT), and IgE^+^ clones corresponding to seven allergen specificities were identified using phage display of single-chain variable fragments (scFv) (45). Blood and nasal mucosal B-cell repertoires were determined by AIRR-Seq, revealing repertoire characteristics similar to those in Wu *et al.* (40), e.g. enrichment of V_H_ mutations in mucosal B cells in allergic subjects, although this could not be attributable to SIT.

Identification of allergen-specific IgE^+^ (scFv) clones that could be mapped to the AIRR-Seq data in the course of SIT (up to 1 year) represented the key strength of this study. The authors demonstrated longitudinal persistence of allergen-specific IgE^+^ clones that displayed molecular features of GC reactions, i.e. expansion, SHM and CSR, among related members. In line with the two aforementioned AIRR-Seq studies, IgE^+^ B cells (allergen specific in this case) were strictly related to hypermutated IgA/IgG; this could be detected in nasal biopsies, strengthening the concept of *in situ* SHM and CSR. Further analysis of multiple biopsies (proximal and distal sampled from same individuals) may further address how/whether the ontogeny of allergen-specific IgE^+^ B cells could be impacted by cell dissemination of within mucosal tissues.

In summary, the observation of allergen-specific B cells with clonal members in different compartments is consistent with GC reactions and mobilization of resident allergen-specific memory B cells. The results of this work reinforce several themes developed in this review and underwrite our conclusions.

## Conclusions

**Fig. 3.**
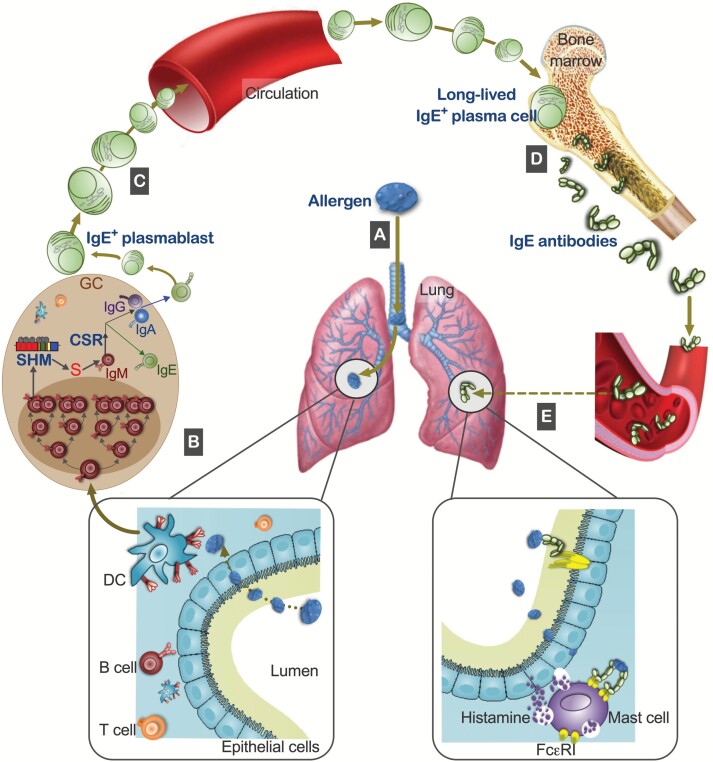
Compartmental regulation and IgE function in allergic asthma. (A) Allergen is inhaled into the lung and travels through the epithelial cell lining of the respiratory tract into the mucosa, where it is captured by dendritic cells (DCs). (B) The DCs and cognate T helper cells induce GC reactions (SHM and isotype switching to IgE) in allergen-specific B cells, causing their rapid differentiation into IgE-secreting cells (Fig. 1). (C) IgE^+^ plasmablasts migrate from local respiratory mucosal tissues through circulation to the bone marrow where they receive survival signals as long-lived plasma cells. (D) Long-lived IgE^+^ plasma cells remain in the bone marrow and secrete IgE antibodies into the blood. (E) The secreted IgE antibodies reach the local respiratory mucosal tissues to bind to mast cells and maintain immediate hypersensitivity.

On the basis of the evidence above, we may draw the following conclusions (Fig. 3):

- The immune memory of IgE responses is embodied in the repertoire of plasma cells contained in the bone marrow.
- IgE antibodies are adapted to equip mast cells for immediate hypersensitivity to antigens/allergens in mucosal tissues.
- This mainly depends on both SHM and CSR to IgE in memory B cells prior to differentiation into IgE^+^ plasma cells.
- Immediate hypersensitivity to aeroallergens occurs in the respiratory mucosa in sensitized individuals.
- The respiratory mucosa is the source of IgE immune memory for aeroallergens in bone marrow in mice.
- The development of IgE^+^ plasma cells *in situ* (in the respiratory tract mucosa) in man is promoted by high levels of SHM.

The entire developmental process of IgE^+^ plasma cells, i.e. the principle of IgE immune memory, may take place in the mucosa itself or adjacent lymphoid tissue. Certain stages may involve the formation of ectopic lymphoid tissue within the mucosa or the activity of constitutive lymphoid tissues adjacent to the respiratory tract mucosa (adenoids, tonsils), followed by homing of memory B cells to the mucosa and plasma cell differentiation. Which event happens precisely where, when and how still remains unclear (46).

## Funding

The authors were supported by a Medical Research Council (MRC) and GlaxoSmithKline Strategic Alliance Programme (Grant number G11002), the National Institute for Healthy Research (NIHR) Biomedical Research Centre (BRC) at Guy’s and St Thomas’ NHS Foundation and King’s College London, the Welcome Trust and the Rosetrees Trust (A1023) and ERDF & HEFCE (G16028 MedCity Collaborate to Innovate). The views expressed are those of authors and not necessarily those of the National Health Service, the NIHR or the Department of Health.

*Conflict of interest statement:* the authors declared no conflicts of interest.
